# Internet Use and Psychological Well-Being at Advanced Age: Evidence from the English Longitudinal Study of Aging

**DOI:** 10.3390/ijerph15030480

**Published:** 2018-03-09

**Authors:** David Quintana, Alejandro Cervantes, Yago Sáez, Pedro Isasi

**Affiliations:** Department of Computer Science, Universidad Carlos III de Madrid, 28911 Madrid, Spain; acervant@inf.uc3m.es (A.C.); ysaez@inf.uc3m.es (Y.S.); isasi@ia.uc3m.es (P.I.)

**Keywords:** aging, Internet, well-being, ELSA

## Abstract

This work explores the connection between psychological well-being and Internet use in older adults. The study is based on a sample of 2314 participants in the English Longitudinal Study of Aging. The subjects, aged 50 years and older, were interviewed every two years over the 2006–2007 to 2014–2015 period. The connection between the use of Internet/Email and the main dimensions of psychological well-being (evaluative, hedonic and eudaimonic) was analyzed by means of three generalized estimating equation models that were fitted on 2-year lagged repeated measurements. The outcome variables, the scores on three well-being scales, were explained in terms of Internet/Email use, controlling for covariates that included health and socioeconomic indicators. The results support the existence of a direct relationship between Internet/Email use and psychological well-being. The connection between the main predictor and the score of the participants on the scale used to measure the eudaimonic aspect was positive and statistically significant at conventional levels (*p*-value: 0.015). However, the relevance of digital literacy on the evaluative and the hedonic components could not be confirmed (*p*-values for evaluative and hedonic dimensions were 0.078 and 0.192, respectively).

## 1. Introduction

Psychological well-being is a complex concept that, according to many studies [1,2,3,4], consists of three major dimensions: evaluative, hedonic and eudaimonic. The first element has to do with the cognitive-judgmental component; the second involves the affective one and includes feelings like happiness or sadness, and the third one covers aspects related to meaning or purpose of one’s life.

The evidence of the connection between the use of Internet and psychological well-being is mixed. While the initial evidence found a clear inverse association [5], further studies found that the connection might not be that strong [6,7] or even non-existent [8]. Conversely, other studies report that Internet use makes a positive and significant contribution to mental well-being [9,10].

Even though there are many studies devoted to younger people [11,12] or the broader population [13], there are fewer focused on older adults that, as [14] mentions, tend to be found on gerontology and human–computer interaction journal publications. Among these, we could mention contributions like [9,10,15,16,17].

The mechanisms by which the use of the Internet could affect psychological well-being are diverse. According to [18], the main psychosocial links between Internet use and mental health in later life are the improvements on interpersonal interaction at the individual level, enhanced access to resources, empowered social inclusion and the society level.

One of the most important ones is the prevention or the mitigation of the effects of social isolation and loneliness, which are emerging risks for older adults [19,20]. Health issues, together with the death of friends and relatives, increase the prevalence of these problems among older people. Given that social networks play an important role in this [21], information technologies in general, and the Internet in particular, have raised a lot of interest, as they are perceived to offer a lot of potential in this context. However, despite all the research on the matter, the evidence on whether Internet-mediated social interaction can effectively mitigate loneliness or social isolation, and hence, affect psychological well-being, is still inconclusive [22].

The recreational use of the Internet has also been identified as a relevant aspect connected to mental well-being among older-adults [16]. Internet use can function in this regard both as a source of entertainment in itself, and a facilitator. According to [23], there might be a two-way connection. Active people might be more prone to explore new activities, including Internet use while, at the same time, Internet use facilitates engagement with other other activities.

Given that the different mechanisms may impact the mentioned three dimensions of mental well-being asymmetrically, the aim of this study is to contribute new evidence regarding connection between Internet use and psychological well-being at advanced age, providing the finer-grained detail that comes with separate analysis for each of the three core aspects of psychological well-being.

The analysis relies on the evidence gathered by the English Longitudinal Study of Aging (ELSA) [24]. The work is a secondary data analysis based on ELSA waves. All of them were granted ethical approval from UK National Health Service Research Ethics Committees, under the National Research and Ethics Service. The study, designed along the lines of [25], is based on panel data analysis by generalized estimating equations (GEE).

## 2. Materials and Methods

### 2.1. Study Population

Data was obtained from the English Longitudinal Study of Aging. This study is a longitudinal survey designed to track the evolution of aging and quality of life among older people. The aim of the study is contributing to a better understanding of what it means to grow older in the 21st century.

The sample covers people aged 50 and over, that live in private households in England. Every two years, it measures a wide range of aspects on a representative sample of people. Among the topics covered by the survey we can mention: household and individual demographics; health; income and assets; social participation; expectations, etc. In addition to these, some waves include one-off modules and questions.

ELSA is regularly refreshed including new samples. This is done to fill the gap left over time at the youngest ages that would, otherwise, leave the category underrepresented. So far, it has been updated at waves 3, 4, 6 and 7. This panel study began in 2002, and it is based on a sample drawn from participants on a previous project, the Health Survey for England (HSE), that took place between 1998 and 2011.

In this particular study we used data from waves 3 to 7 and therefore, the information was gathered every two years from 2006–2007 to 2014–2015. The relevant interviews took place in 2006–2007 (W3), 2008–2009 (W4), 2010–2011 (W5), 2012–2013 (W6) and 2014–2015 (W7).

At wave 3, 9771 individuals were interviewed, but 2814 were removed due incomplete baseline data. There was a large number of participants that was lost to follow up, as only 3547 individuals remained at wave 7, and there was complete information over the 8-year period for 2314.

The factors and covariates were measured at all follow-up interviews with the exception of age, sex, education, wealth and marital status which were only measured at baseline.

Attrition was associated with slightly older individuals, with lower digital literacy, less educated, with a higher degree of functional impairment and lower net wealth.

### 2.2. Measurements

The three aspects of psychological well-being introduced before will be measured using indicators already introduced by Steptoe et al. [26].

- The evaluative component was assessed using the Satisfaction with Life Scale (SWLS) [27]. The scale consists of five statements and 7 possible values that range from 0 (strongly disagree) to 6 (strongly agree). That is, the maximum score, associated with the greatest satisfaction with life, is 30.
- The hedonic dimension of well-being was measured with the Enjoyment of Life Scale, which is obtained from CASP-19 scale [28]. This indicator, closely related to the pleasure scale of CASP-19, and already tested in previous studies [29,30], summarizes the scores of four questions with responses in the range 0 to 3. Higher values indicate higher enjoyment of life.
- The eudaimonic well-being, was evaluated using the questions of CASP-19 that were not used to measure the previous dimension. That included questions related to different aspects of the concept [31] such as control, personal growth, and self acceptance. The score in this scale ranged from 0 to 45 with higher scores indicating greater eudaimonic well-being.

The main predictor was the reply to the answer “I use Internet/E-mail: yes/no”. This proxy for digital literacy was represented with a dichotomous variable. A negative response was encoded with a (0) and a positive with a (1).

Other factors and covariates considered in the models included health and socioeconomic indicators such as:

- Age: classified in four intervals: 50–59 (1), 60–69 (2), 70–79 (3), and 80 and older (4).
- Sex: dichotomous variable that specifies whether the participant is a man (0) or a woman (1).
- Marital status: given that there is evidence supporting that married people tend to report higher well-being [32,33], a dichotomous variable was used to represent whether the subject was married (1) or not (0).
- Education: this variable indicates the highest academic qualification obtained by the subject using a 3-way qualification split. The categories considered were: Less than O-level or equiv (0) O-level or equivalent (1) and higher than a-level (3). These were used to represent no qualification, intermediate qualification or degree or equivalent, respectively.
- Wealth: the relevance of the association of this variable and well-being is supported in previous works like [34]. In this study wealth will be defined as the net non-pension household wealth. This item will be categorized in quintiles, as [35] suggests that relative income might be more important than absolute income, and the variable will be defined in the range 1 to 5. Higher values represent more wealth.
- Lack of impairments: given the importance of this aspect in the independence and quality of life for older people, the study includes a dichotomous variable that indicates whether the person reported (0) or not (1) having suffered any limitation in activities of daily living (ADLs) or instrumental activities of daily living (IADSLs) from emotional, physical, mental or memory problems for more than three months.
- Physical activity. This marker for health behavior summarizes the self-reported physical activity of the subject using four levels in the range 0 to 3. These values represent sedentary, low, moderate and high, respectively. Given that the last waves of the survey replace this summary item with more detailed information, a categorization criterion was required. Following [36], the equivalence was established as: Sedentary: mild exercise 1–3 times a month and no moderate or vigorous activity; Low: mild, but no vigorous activity at least once a week; Moderate: moderate activity more than once a week, or vigorous activity between once a week to 1–3 times a month. Finally the physical activity indicator was considered as high in case it involved heavy manual work or vigorous activity more than once a week. This equivalence is also consistent with [37,38,39].
- Volunteering. This social covariate is also dichotomous, and indicates whether the individual has been involved (1) or not (0) in volunteering activities during the las year. The importance of volunteering in this context has been supported, among others, in [40,41,42].
- Associative interests. This proxy for social connectedness measures the number of different kinds of organizations that the individual reported to be part of. These are grouped into eight broad categories: political party, trade union or environmental groups; tenants groups, resident groups, neighborhood watch; church or other religious group; charitable associations; education, arts or music groups or evening classes; social clubs; sports clubs, gyms, exercise classes; other organizations, club or societies. It should be noted that the indicator summarizes the number of categories, not the number of organizations, and, therefore, it is defined in the 0 to 8 range. The importance of social connectedness in general has been reported, among others, in [42].
- Cognitive ability. This variable was proxied with one of the tests included in the Consortium to Establish a Registry for Alzheimer’s Disease (CERAD) Neuropsychological battery [43]. More specifically, a learning recall test is performed over a delayed word-list. This exercise consists of 10 randomly selected words that the respondents are asked to recall with a 5-min delay. The variable represents the number of correct answers to this test.

### 2.3. Statistical Analysis

The core statistical analysis was based on generalized estimating equations. These models, that extend generalized linear models accounting for within-subject correlations across repeated measurements, are used to measure population-averaged effects over periods of time [44].

For each of the tree components of psychological well-being, a different model was fitted. These models had the scores of the one of the relevant scales, SWLS (evaluative), EOLS (hedonic) EOLS (EDS (eudaimonic), as the independent variables, and digital literacy as the main predictor. In addition to that, the models controlled for all the mentioned covariates: age (stratified); sex; education level; wealth (stratified); marital status; presence of functional impairments; physical activity; results on the delayed recall test; volunteering and the range of types of organizations that subjects reported being members of.

The structure of temporal relation between the predictors and the measures obtained for the well-being indicators was established though 2-year lagged models. The beta regression coefficients and the 95% confidence intervals were obtained using robust covariance estimations, and the statistical significance of the coefficients was tested with the Wald test.

The statistical analysis was performed using SPSS 23 (IBM, Armonk, NY, USA)

## 3. Results

Baseline characteristics of the sample used in the analysis are described in Table 1. There we report the main descriptive statistics: mean, standard deviation, minimum and maximum, for the variables considered in the study. The mean age baseline for the analytical sample was 61.62 ± 7.7 years, and 55% of the subjects were female.

It’s worth noting the disparity in the number of observations observed among the wealth intervals. The difference is explained by the the fact that the assignment to quintiles by wealth was based on the initial Wave 3 dataset, not the filtered analytical sample.

The characteristics of the analytical sample measured at Wave 3 by Internet/Email use, are reported in Table 2. The inspection makes apparent that Internet/Email users tend score higher in the memory test; are more physically active; have a wider array of associative interests, and tend to undertake more voluntary work. Internet/Email users are, on average, more educated and wealthy, and are married more often that non-Internet users. There is a gender bias among non-Internet users, that are more commonly women than men, and age seems to play a role (mean age among Internet/Email users is 65.03 vs. an average of 59.84 among non-users). The scores on the psychological well-being scales are also higher on average among Internet users. The difference, however, is small in relative terms.

Table 3 summarizes the values of the three psychological well-being indicators by main baseline characteristics over the baseline sample. For each indicator, we provide the mean score obtained in the relevant scale, together with the standard deviation, grouped by variable and category. For the number of types of organizations that the subjects reported being member of, and the number of words recalled in the memory test, both discrete variables, we report the means and the standard deviations for the whole sample.

A basis bivariate analysis based on the Wave 3 data shows that Internet/Email users were both the majority and the higher scoring on the well-being scales. There is a clear gradient of wealth on psychological well-being indicators that makes a direct connection between wealth and all happiness dimensions, and that is also the case with the degree physical activity. More active lifestyles are associated to higher psychological well-being across the three dimensions.

Interestingly, age seems to play a different role depending on the dependent variable. While older individuals score higher on average in the scales related to the evaluative and the eudaimonic dimensions, the opposite occurs if one observes the scores on the scales focused on the hedonic one.

The results of the GEE analysis are reported in Table 4. There, for every indicator we report the details of models obtained based on the complete 2006–2014 analytical sample. The fitted models predict the scores on the happiness scales based on the use of Internet/Email and the covariates used as control variables.

The 2-year lagged models are based on the sample described in Section 2.1. As it was discussed, the data gathering effort was made every two years over a period of one year. Participants were not surveyed on the exact same date, but the time point points of successive outcome measures were separated approximately by two years. This regularity made the GEE analysis feasible.

According to the regression coefficients on the models, those individuals who reported not being users of Internet/Email tended to score lower on the three well-being scales. This connection was statistically significant at 5% in one out of the three indicators, the eudaimonic one (*p*-value: 0.015). Having said that, other variables like the degree of physical activity, marital status, suffering functional impairment of wealth turned out to be more relevant.

If we consider the three fully-adjusted models separately, the cognitive-judgmental aspects of well-being, measured with the Satisfaction with Life Scale, have a direct, though marginally significant (*p*-value: 0.078), connection with Internet use; degree of physical activity; volunteering; wealth; lack of impairments related to ADLs or IADSLs; age and being married. Individuals with degrees score higher than those with an intermediate educational level, but subjects in both categories are worse off than those with no education.

The beta coefficients for the enjoyment of life, proxied by EOLS, follow a similar pattern. The main differences are a less clear gradient in relation to age, and the clear statistical significance of the premium obtained by women. In the 2-year lagged model, the positive contribution of Internet/Email use turned out to be limited in absolute terms and statistically insignificant at the conventional levels (*p*-value: 0.192).

The final model, explores the connexion between digital literacy and the eudaimonic aspect of well-being. The sign of the coefficient suggests a direct relation that is also statistically significant at 5% (*p*-value: 0.015, the smallest for Internet/Email use among the three models). The impact of digital literacy, despite of being more modest than the one observed for physical activity, lack of functional impairments or wealth, is still relevant even after controlling for all covariates.

## 4. Discussion

This study contributes with new evidence on the connection between the use of Internet/email and psychological well-being in older adults considering the three main dimensions: evaluative, hedonic and eudaimonic, separately. The results suggest that this approach could be important as, despite of the fact that the signs of the connection between the independent variables and the scores in the tests were the same, positive, the strength and the statistical significance of the connections differed.

In relative terms, the average contribution of Internet/email use to the scores on SWLS and EDS, the instruments related to the evaluative and eudaimonic dimensions, was very similar and higher than to EOLS, focused on the hedonic one. Therefore, the results suggest that, among older community-dwelling population, digital literacy has a positive impact on overall satisfaction with life, and on feelings of control, purpose, or self-acceptance, while the influence on the affective front might be much more limited.

Regarding covariates, the results concur with the findings of previous studies. As expected, there was a clear connection between psychological well-being and volunteering. These include studies like [41], that found it for self-reported happiness, or [42], that report an inverse relationship between volunteering and depression symptoms. The authors of [40] also found a connection between volunteering and positive affect. This was also the case for other variables like wealth [26,35] or physical activity [45].

The results related to the evaluative dimension of psychological well-being are consistent with [10], where authors report a positive relationship between use of Internet and self-reported life satisfaction. There is also precedent in regards to the hedonic dimension, as [9] shows that Internet use has a positive association with mental well-being among retired Americans aged 50 or older, as it reduces the probability of depression very significantly.

Along the same lines, the authors of [16] identified four different Internet uses, and found that all of them had a positive correlation with life satisfaction among a sample of individuals aged 50 years and older. However, after controlling for sociodemographic variables, the positive impact on well-being was significant only for leisure and recreation use. Unfortunately, the present study suffers from limitations in this regard, as we lack the a breakdown of Internet/email use. Hence, we cannot discriminate whether the benefits are coming from the prevention of social isolation, the recreational aspect or some other element.

Another example to illustrate the importance of having detailed information on specific Internet uses would be [46]. There, the analyses lead to the conclusion that there is an inverse relationship between the use of the Internet as a communication tool, and the level or social loneliness. Conversely, the authors report a direct association between the level of emotional loneliness and greater use to find new people. Gaining a deeper understanding of the specific mechanisms by which Internet/Email contributes to each of the three dimensions of psychological well-being requires detailed information on the specific uses.

The authors of [17] provided some insights on the impact over perceived well-being of older adults (65+) of an accessible tablet-based app designed for social connectedness. They report mixed results in terms of self-efficacy. This differs from the results of the present study, where we find a positive impact on the eudaimonic dimension. We wonder whether the fact that their study was conducted on a long-term facility and a retirement home vs. the community-dwelling population considered in ELSA might have introduced a significant bias.

In addition to the descriptions of what Internet/Email was used for, there is a piece of missing information that is likely to be relevant, frequency of use. Some studies suggest excessive use might have a negative impact on psychological well-being [47,48]. Even though Wave 7 of ELSA provides a significantly more detailed picture, unfortunately it will take some time to obtain the information necessary to fit lagged models, which will be explored in future works.

Despite of the limitations, these segmented results contribute to a better understanding of the phenomenon. The scale of ELSA and the use of a national sample makes the reported results generalizable to the population aged 50 years or older not living in assisted living or nursing homes.

The main implication of this study is the need to make separate analysis for each of the three core aspects of psychological well-being. The output of the models support previous evidence on the differential impact of Internet/Email use on the various dimensions. The results also suggest that the study of the mechanisms that connect Internet literacy to the way older-adults experience life purpose, challenges or growth, might be a promising research area with the potential to inspire fruitful intervention programs.

## 5. Conclusions

This study provides new evidence on the connection between the use of Internet/email and psychological well-being in community-dwelling older adults. Unlike other contributions, it considers the three components of the latter separately.

According to the results, the impact on the three dimensions of psychological well-being (evaluative, hedonic and eudaimonic), differs. Even though the signs of the connections between the main predictor and the scores in the tests used to measure them were the same, only the one related to the eudaimonic aspect was statistically significant at conventional levels.

The results are consistent with previous research and the scale of sample used, together with its national scope, makes the reported results generalizable.

## Figures and Tables

**Table 1:** Baseline characteristics of the analytical sample measured at Wave 3. English Longitudinal Study of Aging 2006–2007. Main descriptive statistics.

	Mean	Std. Dev.	Min.	Max.
SWLS Score *	20.49	6.159	0	30
EOLS Score *	10.13	1.624	2	12
EDS Score *	32.94	6.589	6	45
Internet/Email User	0.66	0.475	0	1
Delayed Recall	5.32	1.778	0	10
Physical Activity	2.09	0.719	0	3
Org. membership	1.79	1.410	0	8
Voluntary Work	0.39	0.487	0	1
Sex	0.55	0.498	0	1
Marital Status	0.75	0.431	0	1
Education	1.18	0.807	0	2
Lack of impairments	0.85	0.360	0	1
Wealth Quintile	3.35	1.357	1	5
Age Interval	1.74	0.780	1	4

* Scales used to measure the tree core components of psychological well being: evaluative (SWLS), hedonic (EOLS) and eudaimonic (EDS).

**Table 2:** Baseline characteristics of the analytical sample measured at Wave 3 by Internet/Email use. English Longitudinal Study of Aging 2006–2007. Main descriptive statistics.

	Non-Internet/Email Users				Internet/Email Users *			
	Mean	Std. Dev.	Min.	Max.	Mean	Std. Dev.	Min.	Max.
SWLS Score *	19.77	6.368	0	30	20.86	6.015	0	30
EOLS Score *	9.93	1.781	2	12	10.24	1.526	4	12
EDS Score *	31.20	6.983	8	45	33.59	6.280	6	45
Delayed Recall	4.79	1.748	0	10	5.60	2.989	0	10
Physical Activity	1.97	0.732	0	3	2.16	0.703	0	3
Org. membership	1.52	1.339	0	8	1.93	1.426	0	8
Voluntary Work	0.34	0.224	0	1	0.41	0.492	0	1
Sex	0.62	0.485	0	1	0.51	0.5	0	1
Marital Status	0.68	0.467	0	1	0.79	0.165	0	1
Education	0.78	0.028	0	2	1.40	0.732	0	2
Lack of impairments	0.77	0.419	0	1	0.89	0.319	0	1
Wealth Quintile	2.94	1.345	1	5	3.56	1.315	1	5
Age Interval	2.06	0.821	1	4	1.57	0.701	1	4

* Scales used to measure the tree core components of psychological well being: evaluative (SWLS), hedonic (EOLS) and eudaimonic (EDS).

**Table 3:** Baseline characteristics of the analytical sample by psychological well-being indicator. English Longitudinal Study of Aging 2006–2007.

		SWLS Score *		EOLS Score *		EDS Score *	
	n (%)	Mean	Std.	Mean	Std.	Mean	Std.
Internet/Email User							
No	792 (34%)	19.77	6.368	9.93	1.781	31.20	6.983
Yes	1522 (66%)	20.86	6.015	10.24	1.526	33.59	6.280
Physical Activity							
Sedentary	42 (2%)	16.57	7.979	8.86	1.555	27.51	8.055
Low	373 (16%)	19.34	6.709	9.65	1.750	30.50	7.065
Moderate	1225 (53%)	20.48	5.975	10.12	1.614	32.99	6.331
High	674 (29%)	21.37	5.849	10.49	2.125	34.52	6.099
Voluntary Work							
No	1423 (61%)	19.80	6.438	9.99	1.689	32.34	6.875
Yes	891 (39%)	21.59	5.511	10.35	1.489	33.89	5.988
Sex							
Male	1041 (45%)	20.64	5.934	10.04	1.648	32.85	6.370
Female	1273 (55%)	20.36	6.336	10.12	1.600	33.01	6.765
Marital Status							
Single	572 (25%)	18.05	6.896	9.86	1.659	32.49	6.884
Married	1742 (75%)	21.29	5.676	10.22	1.602	33.08	6.485
Education							
None	579 (25%)	20.11	6.220	9.94	1.699	31.65	6.984
Intermediate	729 (32%)	19.98	6.391	10.02	1.640	32.78	6.639
Degree	1006 (43%)	21.07	5.904	10.22	1.625	33.79	6.183
Lack of impairments							
Yes	354 (15%)	16.64	7.169	9.15	1.806	28.27	7.152
No	1960 (85%)	21.05	5.786	10.31	1.523	33.78	6.115
Wealth Quintile ^†^							
Q1	290 (12%)	17.27	7.517	9.42	1.863	29.48	7.655
Q2	383 (16%)	19.34	6.539	9.80	1.728	31.24	7.075
Q3	479 (21%)	20.38	5.919	10.19	2.307	32.51	6.360
Q4	548 (24%)	21.27	5.393	10.34	1.497	33.73	5.896
Q5	614 (27%)	22.10	5.260	10.43	1.489	35.25	5.379
Age							
50–59	1059 (46%)	19.58	6.559	10.05	1.708	32.74	6.721
60–69	839 (36%)	20.82	5.983	10.20	1.539	33.28	6.719
70–79	385 (17%)	21.01	5.355	10.16	1.577	32.62	6.076
>79	31 (1%)	22.52	4.434	10.03	1.791	34.10	6.156
Org. membership	2314 (100%)	20.49	6.159	10.13	1.624	32.94	6.589
Delayed Recall							

* Scales used to measure the tree core components of psychological well being: evaluative (SWLS), hedonic (EOLS) and eudaimonic (EDS). ^†^ Quintile distribution based on the initial unfiltered sample, not the analytical one. Higher quartiles represent more wealth.

**Table 4:** Generalized estimating equations (GEE) analysis of connection between Internet use and psychological well-being test scores, English Longitudinal Study of Aging 2006–2014.

	SWLS Score *				EOLS Score *				EDS Score *			
	Coef. ^†^	95% Conf. Int.		*p* ^§^	Coef. ^†^	95% Conf Int.		*p* ^§^	Coef. ^†^	95% Conf. Int.		*p* ^§^
		Inf	Sup			Inf	Sup			Inf	Sup	
Internet/Email user (predictor)				0.078				0.192				0.015
No	Ref				Ref				Ref			
Yes	0.367	−0.041	0.776		0.075	−0.037	0.187		0.584	0.113	1.054	
Physical Activity				<0.001				<0.001				<0.001
Sedentary	Ref				Ref				Ref			
Low	0.496	−0.056	1.048		0.274	0.128	0.420		0.970	0.377	1.562	
Moderate	1.023	0.464	1.583		0.570	0.418	0.722		2.139	1.528	2.750	
High	1.448	0.812	2.084		0.804	0.634	0.975		3.153	2.458	3.848	
Voluntary Work				<0.001				0.001				0.001
No	Ref				Ref				Ref			
Yes	0.938	0.606	1.270		0.158	0.068	0.247		0.601	0.233	0.968	
Sex				0.633				<0.001				0.120
Male	Ref				Ref				Ref			
Female	0.098	−0.303	0.499		0.226	0.116	0.336		0.354	−0.092	0.801	
Marital Status				<0.001				<0.001				0.723
Single	Ref				Ref				Ref			
Married	2.250	1.741	2.760		0.253	0.125	0.382		−0.094	−0.618	0.429	
Education				0.001				0.136				0.746
None	Ref				Ref				Ref			
Intermediate	−1.016	−1.556	−0.477		0.066	−0.081	0.214		−0.170	−0.784	0.443	
Degree	−0.737	−1.281	−0.193		−0.061	−0.216	0.094		−0.241	−0.862	0.379	
Lack of impairments				<0.001				<0.001				<0.001
Yes	Ref				Ref				Ref			
No	2.613	2.155	3.071		0.897	0.777	1.016		4.535	4.041	5.029	
Wealth Quintile				<0.001				<0.001				<0.001
Q1 (Poorest)	Ref				Ref				Ref			
Q2	1.102	0.416	1.787		0.202	0.028	0.377		1.282	0.525	2.038	
Q3	1.353	0.672	2.035		0.334	0.160	0.509		1.794	1.030	2.557	
Q4	2.028	1.336	2.720		0.455	0.278	0.631		2.883	2.119	3.647	
Q5 (Wealthiest)	2.766	2.068	3.463		0.463	0.285	0.642		3.611	2.838	4.384	
Age				<0.001				<0.001				0.004
50–59	Ref				Ref				Ref			
60–69	0.551	0.193	0.908		0.183	0.082	0.283		0.679	0.274	1.084	
70–79	1.440	0.989	1.891		0.291	0.163	0.419		0.616	0.096	1.136	
>79	1.845	1.069	2.621		0.186	−0.028	0.399		0.005	−0.829	0.840	
Delayed Recall	0.044	−0.045	0.133	0.332	0.025	0.001	0.048	0.037	0.146	0.052	0.239	0.002
Org. membership	0.095	−0.030	0.220	0.134	0.110	0.077	0.143	<0.001	0.212	0.071	0.353	0.003
Linear trend	13.605	12.506	14.703	<0.001	7.622	7.332	7.912	<0.001	22.579	21.369	23.789	<0.001

* Scales used to measure the tree core components of psychological well being: evaluative (SWLS), hedonic (EOLS) and eudaimonic (EDS). ^†^ Beta regression coefficients estimated through 2-year lagged generalized estimating equations. ^§^ *p* values from Wald tes

## Abbreviations

The following abbreviations are used in this manuscript:

ADL	Activity of daily living
IADSL	Instrumental activities of daily living
ELSA	English Longitudinal Study of Aging
GEE	Generalized Estimating Equations
SWLS	Satisfaction with Life Scale
